# *Onchocerca volvulus* bivalent subunit vaccine induces protective immunity in genetically diverse collaborative cross recombinant inbred intercross mice

**DOI:** 10.1038/s41541-020-00276-2

**Published:** 2021-01-26

**Authors:** Nathan M. Ryan, Jessica A. Hess, Fernando Pardo-Manuel de Villena, Benjamin E. Leiby, Ayako Shimada, Lei Yu, Amir Yarmahmoodi, Nikolai Petrovsky, Bin Zhan, Maria Elena Bottazzi, Benjamin L. Makepeace, Sara Lustigman, David Abraham

**Affiliations:** 1 Department of Microbiology and Immunology, Sidney Kimmel Medical College, Thomas Jefferson University, Philadelphia, PA USA; 2 Department of Genetics, Lineberger Comprehensive Cancer Center, University of North Carolina–Chapel Hill, Chapel Hill, NC USA; 3 Division of Biostatistics, Department of Pharmacology and Experimental Therapeutics, Sidney Kimmel Medical College, Thomas Jefferson University, Philadelphia, PA USA; 4 Flow Cytometry Core Facility, Sidney Kimmel Cancer Center, Thomas Jefferson University, Philadelphia, PA USA; 5 Vaxine Pty Ltd, Flinders University, Bedford Park, SA Australia; 6 Department of Pediatrics, National School of Tropical Medicine, Baylor College of Medicine, Houston, TX USA; 7 Texas Children’s Hospital Center for Vaccine Development, 1102 Bates St, Ste. 550, Houston, TX USA; 8 Institute of Infection, Veterinary & Ecological Sciences, University of Liverpool, 146 Brownlow Hill, Liverpool, L3 5RF UK; 9 Laboratory of Molecular Parasitology, Lindsey F. Kimball Research Institute, New York Blood Center, 310 E 67th St, New York, NY USA

**Keywords:** Protein vaccines, Parasitic infection, Adjuvants, Adaptive immunity

## Abstract

This study tests the hypothesis that an *Onchocerca volvulus* vaccine, consisting of two recombinant antigens (*Ov*-103 and *Ov*-RAL-2) formulated with the combination-adjuvant Advax-2, can induce protective immunity in genetically diverse Collaborative Cross recombinant inbred intercross mice (CC-RIX). CC-RIX lines were immunized with the *O. volvulus* vaccine and challenged with third-stage larvae. Equal and significant reductions in parasite survival were observed in 7 of 8 CC-RIX lines. Innate protective immunity was seen in the single CC-RIX line that did not demonstrate protective adaptive immunity. Analysis of a wide array of immune factors showed that each line of mice have a unique set of immune responses to vaccination and challenge suggesting that the vaccine is polyfunctional, inducing different equally-protective sets of immune responses based on the genetic background of the immunized host. Vaccine efficacy in genetically diverse mice suggests that it will also be effective in genetically complex human populations.

## Introduction

Onchocerciasis, also referred to as river blindness, is a debilitating eye and skin disease caused by the filarial worm *Onchocerca volvulus* and is the world’s second leading cause of infectious blindness. The primary area of endemicity for *O. volvulus* is sub-Saharan Africa, where an estimated 120 million people are at risk of developing onchocerciasis with 20 million infected and 1.2 million suffering from vision impairment or blindness^1^. The life cycle of this parasite begins when an *O. volvulus* infected black fly of the genus *Simulium* takes a blood meal and deposits infective third-stage larvae (L3) into the human host’s dermal tissues. Once in the host, the L3 complete two molts to develop into male and female adult worms that mate and produce microfilariae, which are responsible for transmission and most of the pathology associated with the infection. The microfilariae in the skin of an infected individual are ingested by black flies during a blood meal and develop into L3 to continue the life cycle^2^.

Presently, control of onchocerciasis is through the mass drug administration (MDA) of ivermectin which has a number of challenges preventing complete disruption of transmission within endemic areas. (1) Ivermectin is only effective at killing the microfilariae, requiring annual MDA for 14 years during the reproductive lifespan of the adult female worms^3^. (2) Treatment of patients with ivermectin in several geographic locations resulted in suboptimal microfilaricidal responses^4–6^. (3) Severe adverse reactions may occur if treatment is delivered to individuals that also have high *Loa loa* microfilaremia^7,8^. (4) Non-compliance in taking ivermectin within some endemic populations prevents effective control of transmission^9^. (5) Ivermectin is not recommended for use in children under 5 years of age leaving a large group of individuals untreated and thus creating a reservoir for *O. volvulus* transmission^10^. Therefore, it is critically important that MDA with ivermectin be supplemented with additional intervention tools, including macrofilaricides, vector control^11^ and a prophylactic vaccine for prevention of infection with *O. volvulus* ^12^.

The lack of small animal models for studying *O. volvulus* led to the development of a novel system, in which diffusion chambers containing L3 are implanted subcutaneously into mice. Membranes adhered to the diffusion chamber rings contain the larvae within, while allowing host cells and soluble immune components to traffic freely^13^, thereby providing a unique opportunity to explore how the murine immune components interact with the parasite within its microenvironment. Studies using the diffusion chamber mouse model have demonstrated that immunization with irradiated L3 induced a protective immune response against *O. volvulus* that was dependent on Th2 cytokines, IgE and eosinophils^14^. Vaccination of populations living in endemic regions with attenuated L3 is not technically feasible. To overcome this obstacle, studies were performed to identify subunit vaccine antigens that, when combined with an adjuvant, elicit significant protection against challenge with L3^15,16^. Two recombinant antigens, *Ov-*103 and *Ov-*RAL-2, have been identified as lead candidates for an *O. volvulus* vaccine. When formulated with alum as the adjuvant, these two antigens achieved the greatest reduction in larval survival in BALB/cByJ mice, as compared to other candidate antigens^17^. This observation was confirmed using homologous antigens from the filarial worm *Brugia malayi*. Induction of protective immunity against the L3 infection was accomplished by vaccinating gerbils with alum-adjuvanted *Bm-*103 and *Bm-*RAL-2 antigens individually or in combination^18^.

To increase potency of the *O. volvulus* vaccine in mice, various adjuvant formulations were tested. The result was the selection of *Ov-*103 and *Ov-*RAL-2 with the combination-adjuvant Advax-2^19^ (*Ov* vaccine) as the optimal formulation. The *Ov* vaccine consistently induced significant larval killing through a balanced Th1/Th2 adaptive immune response^20^. A mixed Th1/Th2 cellular response against the infective stage of the parasite appeared to be essential for the protective immunity to *O. volvulus* that develops in putatively immune individuals and in infected individuals who developed concomitant immunity to the infection^21,22^.

All previous pre-clinical development of the *Ov* vaccine was based on experiments done exclusively in BALB/cByJ and C57BL/6J mice. While these studies have established the vaccine’s efficacy and its dependence on humoral and cellular immunity^20,23^, they are limited by the genomic homogeneity within these two inbred strains of mice^24^. The lack of genetic diversity in these animal models may become an issue when the vaccine is advanced to clinical trial in humans, when more diverse host genetics may impact vaccine efficacy^25–28^. Several studies have described the effects of host genetics on *O. volvulus* pathogenesis and disease outcome. Polymorphism in the *IL13* gene caused an increase in serum IL-13 and IgE after exposure to *O. volvulus*, resulting in sowda, a hyper-reactive form of onchocerciasis characterized by increased skin eosinophilia^29^. Furthermore, study of human populations in West Africa linked an HLA-D haplotype to persons that are either immune to reinfection or remain free of infection despite multiple exposures^30^. Therefore, it is important to consider the potential effects that host genetics might have on the efficacy of the *Ov* vaccine.

A panel of genetically diverse mice, Collaborative Cross recombinant inbred strains (CC-RI), was developed using eight founder strains, of which five were classical inbred strains and three were wild-derived inbred strains^31^. CC-RI lines were generated by three generations of intercross breeding using varied combinations of founder strains followed by inbreeding to establish homogeneity within the lines. This resulted in the creation of recombinant mouse lines with greater genetic diversity while retaining reproducibility^32^. To further increase the magnitude of genetic diversity and to test outbred rather than inbred individuals, CC Recombinant Inbred Intercross mice (CC-RIX) were generated through crosses of CC-RI mice resulting in F1 hybrid lines. This design increases diversity when compared to CC-RI, and creates outbred mice with reproducible genomes within CC-RIX lines^33^. The 8 CC-RIX lines chosen for this study were selected to maximize genetic diversity captured by the lines (on average 6.8 haplotypes of a maximum of 8 are present genome-wide), breeding performance, and reproducibility of each line^34^.

The capacity of the *Ov* vaccine to induce elimination of challenge larvae was evaluated in 8 genetically diverse CC-RIX lines. Comprehensive analysis of multiple immune factors, measured systemically and locally at the site of the challenge infection, was performed to determine associations between larval killing and specific immune responses. Protective immunity developed in 7 out of the 8 CC-RIX lines, each through a unique combination of immune responses. The present study demonstrates that the *Ov* vaccine can induce protection against *O. volvulus* infection through multiple unique mechanisms, based on the genetic background of the host, which suggests that the *Ov* vaccine could be successful in protecting heterogeneous human populations from this infection.

## Results

### Protective immunity in genetically diverse CC-RIX lines

**Fig. 1:**
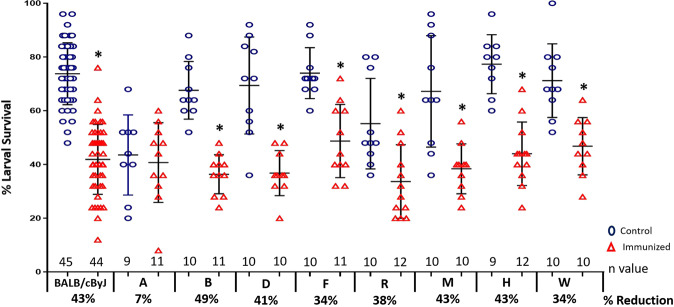
Survival of larval *Onchocerca volvulus* in control and immunized CC-RIX and BALB/cByJ mice. Mice were vaccinated intramuscularly with *Ov*-103 and *Ov*-RAL-2 formulated with Advax-2 (red triangles) while the control mice received only Advax-2 (blue circles). Diffusion chambers containing challenge infections of 25 *O. volvulus* infective third-stage larvae were recovered one-week after implantation and larval survival was determined. Mean percent larval survival ± standard deviations are marked for each group. *n* values for each group are listed above each strain/line, while percent reduction in larval survival comparing vaccinated to controls is listed below each strain/line. *statistically significant, *p* ≤ 0.05, when comparing results from control mice to vaccinated mice within each strain/line.

BALB/cByJ mice and 8 CC-RIX lines were vaccinated with the *Ov* vaccine composed of two antigens, *Ov-*103 and *Ov-*RAL-2, formulated with Advax-2 as the adjuvant. Vaccinated and adjuvant control mice received challenge infections consisting of L3 within diffusion chambers implanted subcutaneously. Vaccinated BALB/cByJ mice and 7 of the 8 CC-RIX mouse lines were protected against L3 challenge as indicated by statistically significant reductions in larval survival ranging from 34%–49% as compared to controls (Fig. 1). Assessing protective immunity based on the mouse gender indicated that, with the exception of vaccinated female mice from Line R, both male and female CC-RIX mice developed vaccine-induced protective immunity at equivalent levels (Supplementary Table 1). Vaccine-induced protection against larvae was not observed in Line A, with survival of the larvae in the adjuvant control group (44%) equivalent to that of the vaccinated mice (41%) and significantly lower than the mean parasite recovery from the adjuvant controls for the other 7 CC-RIX lines (71% ± 14, *p* < 0.001) (Fig. 1).

### Local immune responses to challenge parasites measured in diffusion chambers

Populations of immune cells that migrated to the parasite microenvironment in the diffusion chambers were identified and quantified using flow cytometry. In the control mice from BALB/cByJ and CC-RIX lines exhibiting vaccine-induced protective immunity, the median total number of cells found within the diffusion chambers ranged from 2,966 (Interquartile Range (IQR): 1,208–3,658) cells in Line R to 18,032 (IQR: 11,806–26,022) cells in Line H. Whereas in vaccinated and protected mice, the total number of cells ranged from 1,691 cells in Line R to 36,903 cells in BALB/cByJ (Table 1). The only statistically significant increases in the total number of cells seen in the diffusion chambers recovered from protected mice, as compared to adjuvant controls, was in BALB/cByJ mice and Line D mice. The most abundant cells found within the diffusion chambers for all mice regardless of vaccination status and strain/line were neutrophils and natural killer (NK) cells (Table 1).

**Table 1:** Cell profiles within fluid recovered from diffusion chambers implanted in BALB/cByJ and 8 CC-RIX Lines.

	BALB/cByJ	A	B	D	F	H	M	R	W
**NK Cells**	9433* [2×]	7273	3594	3112* [3×]	1398	2442	1888	396	1699
	(5411, 28,981)	(4579, 13,442)	(392, 84,492)	(2428, 13,948)	(696, 2279)	(1841, 4371)	(1557, 2066)	(206, 819)	(1132, 3739)
**Dendritic Cells**	279	1343	745	636* [4×]	579	535	1023	248	1300
	(188, 525)	(929, 3759)	(104, 23,065)	(445, 1726)	(335, 1156)	(394, 1104)	(527, 1082)	(183, 409)	(880, 2019)
**Neutrophils**	14042* [3×]	11740	1315	11298* [7×]	2133	6204	305	307	4494
	(4134, 43,746)	(6297, 31,143)	(313, 151,125)	(8620, 35,869)	(606, 5372)	(3440, 11373)	(174, 616)	(224, 1365)	(1995, 7593)
**Eosinophils**	440	68	14	148	108	433	86	27	14
	(181, 970)	(41, 136)	(13, 54)	(27, 256)	(54, 136)	(102, 1032)	(66, 122)	(17, 32)	(14, 20)
**Monocytes**	315* [1.4×]	79	122	271	54	165	68	31	67
	(150, 2234)	(54, 150)	(0, 875)	(202, 835)	(27, 135)	(89, 1064)	(33, 135)	(19, 82)	(41, 108)
**Macrophages**	300	914	1043	1279* [9×]	121	837	623	49	1124
	(189, 1050)	(604, 4385)	(13, 1538)	(459, 1845)	(67, 691)	(526, 1320)	(336, 698)	(27, 123)	(807, 2912)
**T Cells**	1480* [2×]	951	678	499	496	777	223	479	284
	(724, 2424)	(590, 1376)	(418, 5551)	(284, 988)	(364, 686)	(660, 983)	(169, 321)	(433, 638)	(264, 902)
**B Cells**	534* [2×]	573	532	406	393	308	95	126	183
	(291, 927)	(302, 1117)	(26, 4183)	(325, 2302)	(175, 638)	(161, 488)	(61, 183)	(56, 362)	(75, 251)
**Total Cells**	36903* [3×]	21687	9154	15384* [5×]	6705	12746	4409	1691	10418
	(17,671, 75,443)	(15,619, 52,068)	(1266, 483,136)	(12,519, 60,147)	(2368, 10,286)	(7575, 24,950)	(3560, 4586)	(1533, 3369)	(8359, 13,193)

Single cell suspensions were prepared from fluid recovered from diffusion chambers, stained using fluorescent antibodies and analyzed by flow cytometry. Median cell counts (number of cells per diffusion chamber) and interquartile ranges for total number of cells and for the 8 different cell types recovered from immunized CC-RIX and BALB/cByJ mice is presented. When a significant difference between control and immunized mice was observed, the fold increase in immunized mice over control mice is provided [×]. *statistically significant, *p* ≤ 0.05, when comparing results from control mice to vaccinated mice within each strain/line.

The bold values indicate the statistically significant values.

**Fig. 2:**
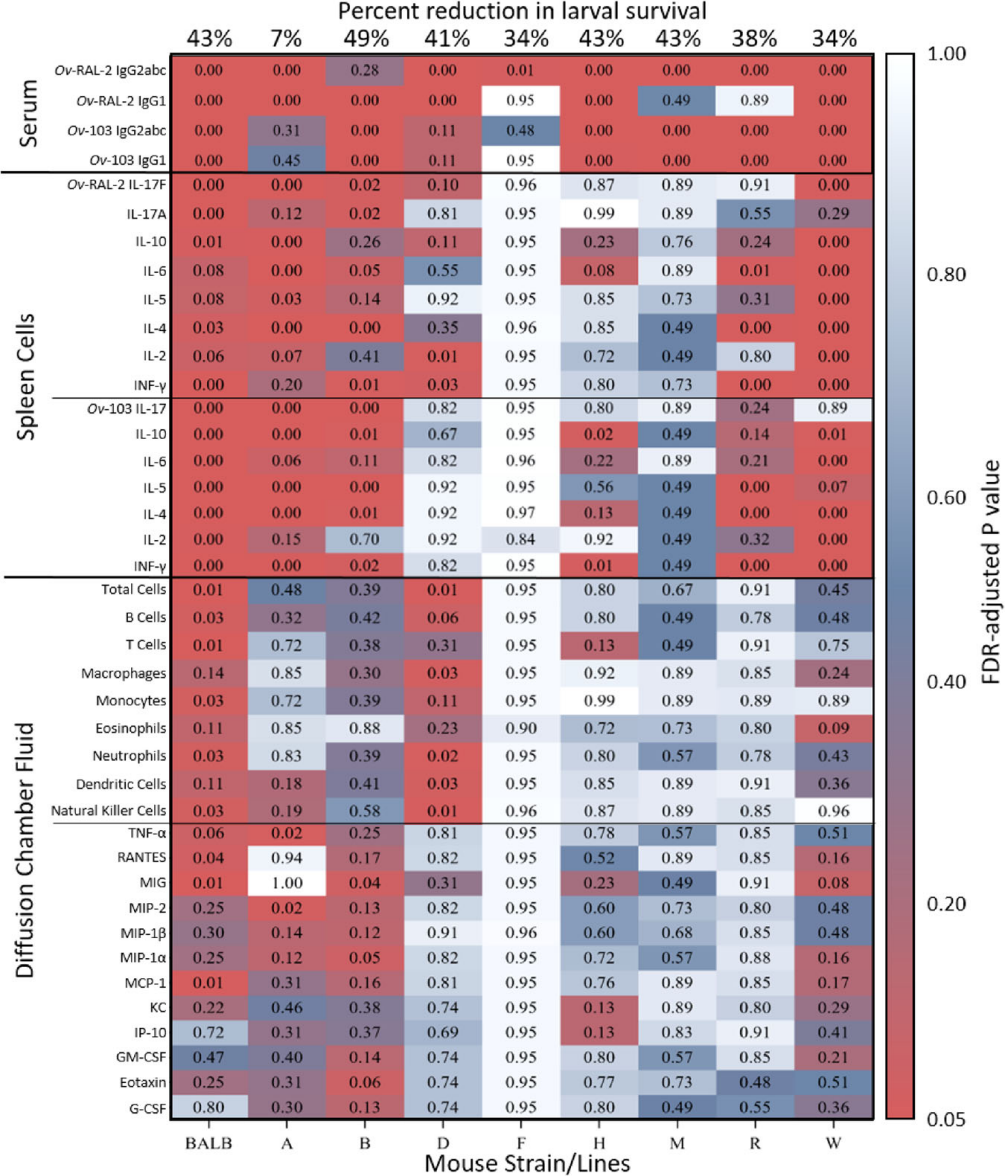
Heatmap summarizing reduction in parasite recovery, and antibody, cytokine, cell recruitment and chemokine responses. Percent reduction in larval survival comparing vaccinated to control mice is presented on the top row for each strain/line. The data collected from all mice from the present study are summarized using Wilcoxon exact tests. *p-*values were calculated using Monte Carlo estimation and false discovery rate (FDR)-controlling procedures to compare each measured immunological factor between control and vaccinated mice following challenge. *p*-Values that approach 1.00 appear in white boxes and progress to blue and then red boxes as they approach a significant *p* ≤ 0.05. **Serum** – measurement in the serum of *Ov*-RAL-2 and *Ov*-103 specific IgG1 and IgG2a/b/c; **Spleen Cells** – measurement of cytokines in supernatant after re-stimulation of spleen cells with *Ov-*RAL-2 and *Ov*-103. **Diffusion Chamber Fluid** – total number and specific cell types as well as measurement of cytokines and chemokines in the parasite microenvironment.

Several cell types were found to be significantly increased in vaccinated BALB/cByJ and Line D mice as compared to control mice, but not in the other CC-RIX lines. In BALB/cByJ, there were significant increases in the number of B cells, T cells, monocytes, neutrophils NK cells. In Line D mice, macrophages, neutrophils, dendritic cells and NK cells were significantly increased (Table 1, Fig. 2). Despite vaccine-induced larval killing in 7 of 8 CC-RIX lines and in BALB/cByJ mice, there were no statistically significant correlations measured between the number of larvae killed in protected mice and either the total number of cells or the number of cells from individual cell types within the diffusion chambers.

To identify soluble immune factors that might be associated with vaccine-induced protection, 11 cytokines and 9 chemokines within the diffusion chamber fluid were measured. All of the 9 chemokines were detected within the diffusion chambers recovered from vaccinated and control mice, however only 3 cytokines, G-CSF, GM-CSF and TNF-α, were present in high enough concentrations to be detected. The only significant increases in median concentration, detected in protected mice as compared to controls, were MCP-1, MIG and RANTES in BALB/cByJ, and MIG and MIP-1α in Line B (Table 2, Fig. 2). None of the levels of cytokines or chemokines that were detected correlated with the number of larval killed, nor were there any cytokines or chemokines that were consistently increased across all protected CC-RIX lines and in BALB/cByJ.

**Table 2:** Cytokine and chemokine profiles in fluid recovered from diffusion chambers implanted in BALB/cByJ and 8 CC-RIX Lines.

	BALB/cByJ	A	B	D	F	H	M	R	W
**G-CSF**	5537	5382	10012	7856	4832	9714	2371	1820	8016
	(3279, 11,773)	(4060, 6003)	(7300, 11,773)	(3292, 11,773)	(3900, 6333)	(3421, 12,992)	(1729, 4277)	(1154, 3250)	(6228, 12,992)
**Eotaxin**	822	71	1033	408	651	224	103	62	64
	(355, 1407)	(36, 231)	(457, 1551)	(258, 562)	(368, 864)	(88, 362)	(78, 201)	(44, 118)	(48, 155)
**GM-CSF**	14	4	20	20	7	12	6	5	21
	(9, 23)	(0, 8)	(8, 30)	(17, 23)	(6, 14)	(7, 39)	(4, 12)	(2, 7)	(12, 27)
**IP-10**	3192	236	1549	1255	179	649	553	436	849
	(2553, 4917)	(202, 307)	(1123, 1722)	(844, 1836)	(138, 392)	(485, 3151)	(428, 892)	(252, 552)	(428, 991)
**KC**	1369	4560	4707	8016	4362	3477	3171	1359	8394
	(637, 2124)	(3503, 5201)	(2997, 5424)	(3640, 11,584)	(1745, 7697)	(1544, 8591)	(2836, 5039)	(989, 2063)	(2218, 12,277)
**MCP-1**	3003* [2×]	1028	5807	4407	929	1062	1300	1487	633
	(1491, 9838)	(509, 1542)	(4441, 8806)	(1537, 6476)	(729, 1293)	(629, 1914)	(1056, 3318)	(575, 1947)	(503, 956)
**MIP-1a**	1753	1693	1654* [2×]	1656	1095	859	528	599	1886
	(1135, 2855)	(1496, 3805)	(919, 9354)	(1051, 4958)	(752, 2503)	(699, 3426)	(328, 734)	(373, 1021)	(772, 3517)
**MIP-1b**	3106	1612	1396	2207	968	962	621	595	1510
	(1785, 14,514)	(1323, 16,586)	(786, 11,230)	(1471, 3799)	(731, 1455)	(475, 14,514)	(300, 763)	(343, 958)	(433, 5730)
**MIP-2**	1273	3518* [3×]	3245	6270	5985	960	1858	776	2650
	(796, 2163)	(2076, 6070)	(1689, 17,551)	(3128, 12,015)	(1607, 6787)	(381, 2349)	(1772, 3057)	(560, 1005)	(1077, 7526)
**MIG**	474* [2×]	15	237* [4×]	64	6	59	16	26	208
	(289, 895)	(10, 47)	(55, 678)	(0, 130)	(3, 14)	(0, 243)	(0, 132)	(6, 49)	(139, 260)
**RANTES**	70* [2×]	4	28	15	0	12	5	3	10
	(26, 131)	(3, 6)	(10, 101)	(8, 19)	(0, 5)	(4, 82)	(3, 9)	(0, 9)	(5, 23)
**TNF-α**	283	602* [3×]	314	626	133	595	215	113	294
	(185, 400)	(346, 775)	(181, 1216)	(214, 1038)	(84, 275)	(229, 1973)	(185, 464)	(76, 181)	(239, 517)

Levels of 11 cytokines and 9 chemokines within diffusion chamber fluid recovered from BALB/cByJ and the 8 CC-RIX lines were analyzed by Luminex. Data shown (median concentrations [pg/ml] with interquartile ranges) are from immunized mice. Data for the 8 cytokines that were not detected have been omitted. *statistically significant, *p* ≤ 0.05, when comparing results from control mice to vaccinated mice within each strain/line. When a significant difference between control and immunized mice was observed, the fold increase in immunized mice over control mice is provided [×].

The bold values indicate the statistically significant values.

Line A, which had low parasite recovery-rates in control mice and the absence of enhanced protective immunity in vaccinated mice, did not have statistically significant changes in cell count within the diffusion chambers for any cell type when comparing vaccinated and control mice. Comparing cytokines and chemokines measured in the diffusion chambers of vaccinated and control Line A mice revealed statistically significant increases in only MIP-2 and TNF-α. Due to the low parasite recovery-rate in control mice of Line A, local immune responses in these mice were compared to the combined control mice from BALB/cByJ and the other 7 CC-RIX lines. Diffusion chambers from control mice from Line A had statistically significant increases in total cells, dendritic cells, neutrophils, macrophages, T cells and B cells when compared to the other control mice (Table 3a). Measurement of chemokines and cytokines from the diffusion chambers demonstrated statistically lower median concentrations of 6 of 9 chemokines and 1 of 11 cytokines while all others remained equivalent between control Line A and all other controls (Table 3b).

**Table 3:** Comparison between immune factors measured in Line A control mice compared to levels measured in CC-RIX and BALB/cByJ control mice.

(a) Diffusion chamber cell count			(b) Diffusion chamber cytokines and chemokines			(c) Spleen cell supernatant cytokines			
Variable	NOT A (*n* = 114)	A (*n* = 9)	Variable	NOT A (*n* = 114)	A (*n* = 9)	Variable		NOT A (*n* = 114)	A (*n* = 9)
Dendritic Cells	346	1211*↑	Eotaxin	471	186*↓	Ov-103	IL-2	31	14*↓
	(122, 650)	(804, 1665)		(184, 872)	(102, 282)			(14, 59)	(12, 15)
Neutrophils	2377	24209*↑	GM-CSF	12	6*↓		IL-4	66	29*↓
	(836, 7723)	(13,302, 33,595)		(6, 19)	(4, 9)			(22, 282)	(10, 45)
Macrophages	230	2115*↑	IL-9	169	99*↓		IL-5	8	0*↓
	(66, 840)	(873, 3562)		(103, 223)	(81, 118)			(0, 29)	(0, 0)
T Cells	490	1051*↑	IL-10	40	12*↓		IL-6	160	695*↑
	(338, 847)	(694, 1187)		(15, 78)	(5, 13)			(61, 338)	(423, 971)
B Cells	242	830*↑	IP-10	1137	313*↓	Ov-RAL-2	INF-γ	680	4920*↑
	(95, 528)	(464, 1174)		(455, 2968)	(227, 477)			(160, 1954)	(2299, 7327)
Total Cells	7186	36068*↑	MIG	119	15*↓		IL-2	44	19*↓
	(3758, 16,451)	(21,570, 50,045)		(9, 255)	(12, 19)			(19, 85)	(14, 30)
			RANTES	9	4*↓		IL-5	24	2*↓
				(4, 31)	(3, 4)			(0, 61)	(0, 6)

Median cell counts (number of cells per diffusion chamber (a)), levels of cytokines and chemokines (pg/ml) in the diffusion chamber fluids (b), and cytokine levels in ex vivo restimulated splenocytes (c) with corresponding interquartile ranges in control mice from Line A and control mice from the other CC-RIX and BALB/cByJ are presented. The data presented are only for factors with statistically significant differences. (*↑) = statistically significant increase in Line A compared to combined control mice from all other groups (NOT A); *p* ≤ 0.05. (*↓) = statistically significant decrease in Line A compared to NOT A control mice; *p* ≤ 0.05.

The bold values indicate the statistically significant values.

### Systemic immune responses

Systemic immune responses in protected mice were evaluated by measuring 14 cytokines in the supernatants of spleen cells following re-stimulation with the antigens *Ov-*103 or *Ov*-RAL-2 ex vivo. Protected BALB/cByJ and Lines B, R and W had increases in Th1, Th2 and Th17 cytokines in response to either *Ov-*103 or *Ov*-RAL-2. When comparing protected and control mice, Line D had a significant increase in one Th1 cytokine after stimulation with *Ov*-RAL-2, while Lines F and M did not have any measurable cytokine responses to either antigen. The cytokines IL-13, IL-17E, IL-21, IL-23, IL-27 and IL-33 were not detected in any of the stimulated splenocyte samples collected from control or protected mice. The absolute quantity of cytokine produced varied for each cytokine, for each strain/line and for each antigen. Furthermore, the fold-increase in cytokine levels produced by cells from protected mice compared to control responses varied greatly, ranging from a less than 2-fold increase for many cytokines to a 39-fold increase for IFN-γ with spleen cells from Line W stimulated with *Ov*-103. The data show that the *Ov* vaccine elicits a mixed cytokine response after infection within the spleen, however, the data from Lines D, F and M suggest that a strong cytokine response is not required for protective immunity (Table 4, Fig. 2). None of the levels of cytokines that were detected in the restimulated spleen cell supernatants correlated with the number of larvae killed, nor were there any cytokines that were consistently increased across all protected CC-RIX lines and in BALB/cByJ.

**Table 4:** Cytokine profiles in supernatants from ex vivo antigen-stimulated spleen cells recovered from BALB/cByJ and 8 CC-RIX Lines.

	BALB/cByJ	A	B	D	F	H	M	R	W
*Ov-*103									
Th1									
INF-γ	1753* [6×]	5021* [4×]	5381* [2×]	2723	1454	3246* [10×]	645	2321* [17×]	1664* [39×]
	(893, 2915)	(4599, 7327)	(3655, 39022)	(450, 18341)	(1026, 2969)	(677, 4271)	(226, 877)	(972, 4264)	(535, 2792)
IL-2	73* [1.4×]	22	31	53	13	21	30	25	39* [5×]
	(50, 110)	(18, 31)	(17, 33)	(43, 147)	(8, 28)	(14, 27)	(18, 37)	(19, 31)	(26, 41)
Th2									
IL-4	168* [3×]	789* [27×]	1446* [3×]	1443	270	41	106	375* [5×]	175* [8×]
	(107, 284)	(625, 921)	(1068, 3124)	(772, 1581)	(197, 360)	(12, 511)	(53, 171)	(204, 808)	(175, 125)
IL-5	90* [9×]	39* [39×]	104* [5×]	7	6	6	13	52* [6×]	8
	(38, 159)	(13, 144)	(73, 115)	(0, 66)	(0, 13)	(0, 15)	(10, 34)	(32, 76)	(6, 10)
IL-6	326* [2×]	2405	686	371	520	108	114	285	548* [8×]
	(173, 531)	(1636, 2951)	(502, 1782)	(170, 860)	(403, 782)	(17, 474)	(89, 272)	(206, 507)	(219, 845)
IL-10	31* [31×]	133* [7×]	55	138	255	54* [3×]	26	69	21* [21×]
	(14, 44)	(98, 210)	(30, 138)	(32, 179)	(184, 326)	(36, 155)	(19, 39)	(35, 180)	(21, 28)
Th17									
IL-17F	84* [84×]	51* [51×]	68* [68×]	0	19	0	0	0	0
	(32, 209)	(28, 72)	(54, 248)	(0, 0)	(0, 53)	(0, 0)	(0, 0)	(0, 24)	(0, 0)
*Ov-*RAL-2									
Th1									
INF-γ	2322* [3×]	4636	3678* [2×]	5955* [2×]	3200	630	619	3383* [60×]	3711* [17×]
	(1100, 4062)	(4046, 5806)	(2848, 4497)	(3607, 39022)	(2735, 4571)	(0, 7393)	(308, 1013)	(2042, 6933)	(1227, 4366)
IL-2	87	57	32	38	40	16	39	26	42* [5×]
	(59, 131)	(20, 94)	(27, 52)	(10, 68)	(21, 46)	(13, 54)	(32, 40)	(0, 31)	(34, 52)
Th2									
IL-4	281* [2×]	631* [5×]	840* [2×]	1239	426	65	205	561* [6×]	552* [13×]
	(80, 448)	(559, 729)	(712, 1050)	(926, 1405)	(251, 568)	(24, 247)	(88, 568)	(293, 862)	(400, 1325)
IL-5	72	33* [17×]	33	26	17	16	20	31	35* [35×]
	(18, 153)	(19, 74)	(24, 134)	(9, 49)	(10, 28)	(12, 22)	(11, 105)	(23, 37)	(21, 82)
IL-6	483	1572* [2×]	842* [2×]	797	1068	1347	347	663* [7×]	4158* [30×]
	(223, 1063)	(1425, 1932)	(468, 1659)	(557, 1796)	(915, 1361)	(115, 2024)	(104, 833)	(377, 1195)	(3260, 5516)
IL-10	54* [2×]	140* [3×]	51	218	374	101	49	63	99* [99×]
	(28, 125)	(109, 204)	(22, 96)	(101, 428)	(282, 452)	(55, 159)	(18, 165)	(42, 82)	(55, 114)
Th17									
IL-17A	123* [123×]	34	234* [2×]	0	95	0	0	0	0
	(0, 294)	(0, 68)	(163, 554)	(0, 15)	(0, 191)	(0, 0)	(0, 0)	(0, 73)	(0, 140)
IL-17F	43* [3×]	28* [28×]	89* [2×]	0	109	0	0	0	16* [16×]
	(14, 142)	(14, 44)	(77, 150)	(0, 3)	(25, 146)	(0, 0)	(0, 0)	(0, 0)	(0, 107)

Levels of 14 cytokines in culture supernatants from spleen cells stimulated ex vivo with *Ov*-103 or *Ov*-RAL-2 were analyzed by Luminex. Data shown (median concentrations [pg/ml] with interquartile ranges) are from immunized mice. Data for the 6 cytokines that were not detected have been omitted. (*) = statistically significant, *p* ≤ 0.05, when comparing results from control mice to vaccinated mice within each strain/line. When a significant difference between control and immunized mice was observed, the fold increase in immunized mice over control mice is provided [×]. A non-detectable result was replaced by ‘1’ in the analysis so that the fold difference could be calculated.

The bold values indicate the statistically significant values.

The role of antigen-specific antibody in the protective immune response was evaluated by measuring *Ov*-103- and *Ov*-RAL-2-specific IgG1 and combined IgG2a, IgG2b and IgG2c (IgG2a/b/c) antibody endpoint titers from serum collected 7 days post-challenge. Immunized and challenged BALB/cByJ mice and all 8 CC-RIX lines developed antibody responses to at least one of the antigens, with the responses from each strain/line having its own unique antibody profile. Antibody responses in mice demonstrating protective immunity following vaccination included: (a) BALB/cByJ mice and Lines H and W had elevated IgG1 and IgG2a/b/c responses specific to both antigens. (b) Lines B, M, and R had an antigen-specific antibody response to both antigens but not with both subclasses. (c) Line D had an *Ov-*RAL-2 response consisting of both IgG1 and IgG2a/b/c but no response to *Ov*-103. (d) Line F only had significant increase in *Ov-*RAL-2-specific IgG2a/b/c. All of the protected strain/lines, except for Line B, had a significant *Ov-*RAL-2-specific IgG2a/b/c response (Table 5, Fig. 2). No statistically significant correlations were found between antigen-specific antibody titer and the level of larval killing.

**Table 5:** Antigen-specific antibody endpoint titers measured in BALB/cByJ and 8 CC-RIX Lines serum following vaccination and challenge.

	BALB/cByJ	A	B	D	F	H	M	R	W
***Ov*-RAL-2**									
IgG2a/b/c	4815*	1668*	899	250,360*	1271*	24,666*	2421*	1061*	223,142*
	(1806, 36,389)	(255, 10051)	(274, 18,622)	(169,248, 536,469)	(101, 5018)	(4884, 118,197)	(221, 2917)	(290, 2050)	(100,586, 564,381)
IgG1	55443*	1527*	688*	98782*	31	89325*	170	28	504425*
	(12,809, 173,732)	(303, 9022)	(113, 9200)	(5072, 402,417)	(14, 562)	(2308, 370,907)	(0, 877)	(0, 127)	(265,933, 927,679)
***Ov-*103**									
IgG2a/b/c	125*	0	4061*	16	13	933*	559*	5107*	110*
	(3, 1305)	(0, 13)	(569, 879,079)	(5, 58)	(0, 367)	(281, 8152)	(208, 2330)	(611, 28,230)	(9, 45,977)
IgG1	1978*	0	41*	0	0	81*	1245*	4197*	4878*
	(131, 148,00)	(0, 24)	(5, 159,808)	(0, 82)	(0, 14)	(15, 1327)	(733, 4205)	(1136, 17,846)	(40, 48,228)

Antigen-specific (*Ov*-103 or *Ov-*RAL-2) and IgG subtype (IgG1 or a combination of IgG2a, IgG2b and IgG2c [IgG2a/b/c/]) endpoint titers were determined using ELISA on serum collected from vaccinated BALB/cByJ and CC-RIX following recovery of challenge larvae. Median endpoint titers with interquartile ranges are presented. *statistically significant, *p* ≤ 0.05, when comparing results from control mice to vaccinated mice within each strain/line.

The bold values indicate the statistically significant values.

Mice in Line A, which did not demonstrate vaccine induced protective immunity, had statistically significant increases in Th1, Th2 and Th17 responses to *Ov-*103 and significant increases in Th2 and Th17 responses to *Ov-*RAL-2 when comparing control and vaccinated mice (Table 4). Antibody responses in vaccinated Line A were similar to mice from Line D, both having a statistically significant increases in *Ov-*RAL-2-specific IgG1 and IgG2a/b/c antibody titers (Table 5, Fig. 2). Stimulated spleen cell cytokine responses from control mice in Line A compared to combined control mice from the 7 other CC-RIX lines and BALB/cByJ were significantly increased for IL-6 in response to *Ov-*103 and for IFN-γ to *Ov-*RAL-2, while demonstrating statistically significant decreases in several other cytokines (Table 3c).

## Discussion

A significant reduction in *O. volvulus* larval survival was observed in 7 of the 8 CC-RIX lines and in BALB/cByJ mice that were vaccinated with *Ov* vaccine, a bivalent vaccine composed of *Ov-*103 and *Ov-*RAL-2 antigens and formulated with Advax-2 adjuvant. These outcomes clearly validate the effectiveness of the *Ov* vaccine at inducing protective immunity in a wide range of genetically-diverse mice. Comprehensive analyses including larval survival, local immune responses measured in the diffusion chambers and systemic immune responses measured in the re-stimulated spleen cells and serum revealed that each strain/line of mice developed a unique combination of the measured immune factors. There were no statistically significant correlations between individual or groups of immune factors and extent of larval killing, either across all protected mice or within each individual strain/line, that could serve as an indicator of the mechanism of protective immunity. The inability to identify correlations may be explained by the limited variation in parasite recoveries observed within the vaccinated and protected mouse lines and their associated highly variable immune responses. There was no single or group of immune factors that were consistently elevated across all protected mice, suggesting that each strain/line of mice developed protection through a unique mechanism.

Previous studies of protective immunity induced by the antigens *Ov-*103 and *Ov-*RAL-2 adjuvanted with Advax-2 utilized a co-administration protocol in which the antigens were injected individually into the mice at a single time point. BALB/cByJ mice immunized with the co-administered antigen vaccine developed protective immunity that resulted in a 47% reduction in the mean larval survival^20^. A similar reduction in larval survival (43%) was observed in this study where BALB/cByJ mice were vaccinated with the *Ov* vaccine injected as one inoculum consisting of both antigens formulated with Advax-2. Importantly, 7 of the 8 vaccinated CC-RIX lines were also protected with a similar 34%–49% reduction in larval survival. These observations support the conclusion that the *Ov* vaccine can induce adaptive protective immunity regardless of the genetic background of the host. Furthermore, the vaccine’s efficacy was equal in both male and female mice, except in Line R. These studies provide evidence that the *Ov* vaccine has the potential to be effective in the heterogeneous human populations living in endemic regions for onchocerciasis.

The *Ov*-103 and *Ov*-RAL-2 vaccine antigens induced consistent levels of protective immunity, resulting in killing of approximately 40% of challenge larvae, regardless of route of administration^17^, adjuvant and formulation^20^, and as shown in this study, host gender, host genetic background, or the type and magnitude of the immune response. These observations suggest that the absence of complete elimination of larvae in the immunized mice was not due to a deficiency in the immune response, but rather due to changes in susceptibility of the larvae as they develop from L3 to fourth-stage larvae (L4). L3 begin to molt into L4 starting on day 3 post-infection^13^ and the surface of the synthesized L4 cuticle was shown to have its own unique characteristics in *O. volvulus* and other filarial worms^35,36^. In the present study, the diffusion chambers were implanted for one week, whereas in previous studies using the co-administered antigens, they were implanted for three weeks^17,20^. Despite allowing more time for the immune response to kill the worms, the same level of parasite killing was observed. Thus, it appears that some worms within diffusion chambers are able to evade the immune killing process during the three weeks in mice. It is possible, that if worm development was allowed to continue, their changing surfaces might have resulted in a renewed susceptibility to immune-mediated killing. Alternatively, developing parasites may boost the vaccine-induced immune response resulting in continued parasite killing. Support for this hypothesis can be found in the report where dogs, immunized against *Dirofilaria immitis* with chemically-abbreviated infections, killed 63% of larvae at 3-weeks post-challenge when contained within diffusion chambers, but 98% of the challenge parasites were killed at 7 months when the adult parasites were recovered from the infected tissues^37^. Further support for this hypothesis can also be found in the studies in which gerbils were vaccinated with *B. malayi* homologous antigens *Bm-*103 and *Bm-*RAL-2 and numbers of adult worms recovered after 90 days post challenge were reduced by approximately 60%^18^. Thus, the *Ov* vaccine could be significantly more effective when the immune response has a longer time to kill the various parasite stages during their development in the host and/or when the worms are within their niche, subcutaneous tissues, as compared to when they are contained within diffusion chambers for a limited time period. Modeling studies concluded that an initial vaccine prophylactic efficacy of 50% and an initial therapeutic efficacy of 90% would markedly reduce microfilarial load in the young age groups protecting them from the morbidity and mortality associated with onchocerciasis. Most benefit would be gained from a long-lived vaccine even if only partially protective^38^. However, it must be emphasized, that a 40% reduction in larval survival would still have a meaningful clinical benefit, translating into a significant reduction in the number of viable adults that colonize the host tissues and thus reducing the number of microfilariae they produce. Microfilarial loads have been directly linked to the pathology caused by *O. volvulus*^39,40^ and a reduction in microfilariae in the skin would also limit transmission of the disease.

It is clear that each CC-RIX line developed a unique immune response following immunization with the *Ov* vaccine and challenge, and that in 7 out of 8 CC-RIX lines and BALB/cByJ mice this multifactorial adaptive immunity resulted in a significant killing of the challenge larvae. The one exception was Line A where parasite recovery in the control and vaccinated mice was equal despite strong Th1, Th2 and Th17 cytokine responses in the stimulated spleen cells and IgG2a/b/c response to *Ov-*RAL-2 in the vaccinated and challenged mice. Parasite recoveries from control mice from Line A were at equivalent levels to that seen in the protected mice from the other CC-RIX lines and BALB/cByJ, suggesting that Line A control mice had an inherent reduction in susceptibility to infection with *O. volvulus* larvae. This hypothesis is supported by the observation of highly elevated numbers of dendritic cells, macrophages, neutrophils, T cells, B cells and total cells found in the diffusion chambers recovered from Line A control mice after challenge as compared to control mice from the other strains/lines. While the cell numbers increased in Line A control mice, there was a concurrent decrease in chemokine levels within the diffusion chamber fluid. This observation might be explained by an increase in the rate at which chemokines from Line A diffused out from the diffusion chamber, resulting in an accelerated rate of cell chemotaxis up the chemokine gradient leading to the worms within the diffusion chamber.

Analysis of the cell populations migrating into the diffusion chambers showed a large degree of variability between the strain/lines and individual mice. These findings limited the possibility of identifying statistically significant changes in cell recruitment into the parasite microenvironment that correlated with parasite survival, across either all of the protected mice or within each individual strain/line, after 7 days in vivo. Previous studies analyzing cell populations in several CC-RIX lines have reported wide variation in the number of T cell, B cell and antigen presenting cells^41,42^, supporting the observations of the present study. Neutrophils and eosinophils have been implicated in the mechanism of protective immunity to *O. volvulus*^20^, which is consistent with neutrophil and eosinophil content in the diffusion chambers measured in both control and immunized mice in the present study. Furthermore, human monospecific anti-*Ov*-103 antibodies but not anti-*Ov*-RAL-2 significantly inhibited the molting of L3 in vitro in collaboration with human neutrophils, while both anti-*Ov*-103 and anti-*Ov*-RAL-2 antibodies significantly inhibited the molting of L3 in the presence of human monocytes^23^. In previous studies, in which diffusion chambers were implanted for three weeks, significant increases in chemokines within diffusion chambers of the protected mice were recorded^17,20^. However, similar findings were not observed in the present study where the diffusion chambers were implanted for only one week. Diffusion chambers represent a dynamic environment in which cells are presumed to be actively migrating into the diffusion chamber while soluble factors diffuse into and out of the parasite microenvironment. Flow cytometry of diffusion chamber contents only provides a single snapshot of the host response that may reflect the environment either during or after the killing process, with each phase having its own unique composition of infiltrating cells and factors.

Cytokine analysis of supernatants recovered from ex vivo stimulated spleen cells showed that the profile of cytokine secretion varied based on mouse genetic background with some lines having a multiple cytokine response to vaccination and challenge and other lines having no significant cytokine response. Immunized and protected mice from BALB/cByJ and Lines B, R and W had increases in Th1, Th2 and Th17 cytokines in response to both antigens whereas Lines D, F, H and M had minimal or no measurable cytokine responses to either antigen. Moreover, the absolute quantity of each cytokine produced by the stimulated spleen cells varied for each strain/line and for each vaccine antigen. Previous studies using BALB/cByJ mice, vaccinated with co-administered *Ov*-103 and *Ov*-RAL-2 each formulated with Advax-2, resulted in the induction of a balanced Th1/Th2-type immunity similar to the outcomes in the present study, when the *Ov* vaccine was administered as one inoculum^20^. Notably, putatively immune individuals and infected individuals who developed concomitant immunity to *O. volvulus* with age also displayed a mixed Th1/Th2 response in response to L3 and molting L3 extracts^21,22^. Balanced Th1/Th2 immunity has also been shown to be associated with the development of protective immunity to *Wuchereria bancrofti* and *B. malayi* vaccine antigens^43,44^. It is clear the *Ov*-103 and *Ov*-RAL-2 antigens in the *Ov* vaccine can induce Th1, Th2 and Th17 cytokine responses based on the cytokine profiles of ex vivo re-stimulated spleen cells recovered from the protected mice. However, the lack of a robust cytokine response in several of the CC-RIX lines while simultaneously having a strong protective immune response, suggests that strong cytokine responses may not be required for protective immunity to larval *O. volvulus*.

Protective immunity induced in C57BL/6J mice by vaccination with either *Ov-*103 or *Ov-*RAL-2 with Advax-2, has been shown to be IgG-dependent^23^. In the present study, antigen-specific antibody responses in the protected BALB/cByJ and CC-RIX mice demonstrated that the vaccine induced both IgG1 and/or IgG2a/b/c responses to either one or both of the vaccine antigens. Analysis of IgE was not included in the antibody analyses, as previous studies in which BALB/cByJ mice were immunized with *Ov*-103 or *Ov-*RAL-2 with Advax-2 did not observe antigen specific IgE responses^20^. The varied responses across the 7 protected CC-RIX lines suggest that the antigen-specific antibody responses following immunization might be controlled by host genetic background. These findings are consistent with other studies that have demonstrated genetic control of antibody responses in mice^45,46^ and in humans^47,48^. A mixed IgG1 and IgG2a/b/c response to both vaccine antigens was observed in protected BALB/cByJ, consistent with previous studies with the co-administered vaccine^20^, and was also observed in Lines H and W. However, a similar level of protective immunity was observed in Lines that only responded to *Ov-*RAL-2, where Line F only developed antigen-specific IgG2a/b/c and where Line D only developed IgG1 and IgG2a/b/c to the antigen. It can be concluded from these antibody response assays that although the *Ov* vaccine is composed of two antigens not all CC-RIX lines responded similarly to vaccination by both antigens. Furthermore, there was a large range in the antibody endpoint titers in the protected CC-RIX mice, with no apparent correlation between antibody titer and efficacy of the killing response within the diffusion chamber. Thus, a varied antibody response, in terms of IgG subclass, antigen specificity and endpoint titer, is also associated with equal levels of protective immunity. This diversity of functional antibody responses portents well for the efficacy of the *Ov* vaccine as it is translated into clinical use.

The vaccine formulation used in this study was selected to maximize the potential of the vaccine to be effective in a wide range of host genetic backgrounds. The *Ov* vaccine is composed of two vaccine antigens that can independently induce protective immunity, and when combined act synergistically to induce a higher level of protective immunity^20^. Combination adjuvants have been shown to enhance vaccine efficacy^19,49^ and the combination adjuvant Advax-2 which contains a polysaccharide particle (delta inulin)^18^ together with a TLR9 agonist (CpG oligonucleotide) was selected as it was shown to induce a mixed Th1 and Th2 response when used together with *Ov*-103 and *Ov*-RAL-2^20^. It was hypothesized that vaccinating mice with two unique antigens, that could function independently, and with an adjuvant that induces a combined Th1 and Th2 response, would enhance the probability that the vaccine would function effectively within a spectrum of genetic backgrounds. Results from the present study confirmed this hypothesis based on the observation that protective immunity developed in 7 out of 8 CC-RIX lines, despite some lines only responding to one of the two antigens in the *Ov* vaccine. Furthermore, most of the lines developed combined Th1, Th2 and Th17 immune responses although there were lines with dominant Th1 response and others with uniformly weak cytokine responses. Importantly, this wide range of cytokine responses to the *Ov* vaccine all resulted in equivalent levels of protective immunity to the infection.

It is clear from Fig. 2 that there was an extensive range of immune responses to the *Ov* vaccine in the protected BALB/cByJ and CC-RIX lines, ranging from multi-component to single-analyte responses, all resulting in the same level of protective immunity. The remarkable variability in the innate and adaptive immune responses between the genetically diverse mouse strain and lines suggests that the *Ov* vaccine is immunologically polyfunctional in nature and capable of inducing multiple protective mechanisms. Alternatively, *Ov* vaccine-induced protective immunity may function in CC-RIX mice through a single conserved mechanism that was not identified in this study. Genetics have been shown to play a critical role in the rate of success for a variety of vaccines including those for measles^27^, influenza^25^ and hepatitis B^26,28^ and are widely believed to be responsible for the variable responses in other vaccines^50^. The multi-facetted responses and broad protection across genetically diverse mouse populations observed in this study may mirror observations of the yellow fever vaccine, YF-17D, in humans. A single dose of YF-17D can induce an immune response consisting of cytotoxic T lymphocytes, balanced Th1/Th2-type immunity and neutralizing antibodies that can last up to 30 years. This polyfunctional response consisting of multiple mechanisms of immunity was responsible for the ability of this vaccine to induce strong protective immunity throughout diverse human populations^51^, despite the high degree of variation observed in antigen specific CD8^+^ T cell responses and neutralizing antibody titers^52^. The findings with YF-17D support the hypothesis that the polyfunctional *Ov* vaccine would be optimal for use in humans.

This study demonstrated that the *Ov* vaccine is capable of inducing protective immunity in genetically-diverse mouse lines. Comprehensive analysis of effector cell recruitment, cytokine and chemokine concentrations within the parasite microenvironment, antigen-specific cytokine and antigen-specific serum antibody responses have supported the conclusion that there is a genetic basis for the type of innate and adaptive protective immune responses induced by the *Ov* vaccine. It was hypothesized that the vaccine could potentially induce multiple killing mechanisms, and in this study, we show that each CC-RIX line uses its own unique combination of immune factors to eliminate the parasites. Future studies are required to further define specific biomarkers of vaccine efficacy and to dissect the mechanisms of the protective immune response. The results from this study show that the *Ov* vaccine functions in a wide range of host genetic backgrounds and suggest that the vaccine will translate effectively into clinical use, protecting diverse human populations from infection with *O. volvulus* through a variety of effective protective mechanisms.

## Materials and methods

### Source of parasites

*O. volvulus* L3 were isolated from newly emerged adult black flies (*Simulium damnosum*) that fed on consenting infected donors (Protocol 320, approved by the New York Blood Center and the Medical Research Station, Kumba, Cameroon IRBs). Flies were kept in a controlled insectary, dissected after one week and the developed L3 were collected, cleaned and cryopreserved as previously described^53^.

### Source of mice

Male BALB/cByJ mice were obtained from The Jackson Laboratory (Bar Harbor, Maine). Male and female CC-RIX mice were bred at the System Genetics Core Facility at University of North Carolina (UNC), Chapel Hill^54^. Sixteen different CC-RI lines were selected as parental lines and then assigned to 8 unique breeding pairs, with each pair comprised of two CC-RI lines. The following 8 CC-RIX lines were generated with the dam listed first and the sire listed second, each given a random code letter to ease analyses: Line A – CC004/TauUnc x CC071/TauUnc; Line B – CC005/TauUnc x CC001/Unc; Line D- CC019/TauUnc x CC055/TauUnc; Line F – CC039/Unc x CC003/Unc; Line H – CC051/TauUnc x CC049/TauUnc; Line M – CC042/GeniUnc x CC007/Unc; Line R – CC040/TauUnc x CC002/Unc and Line W – CC026/GeniUnc x CC006/TauUnc. All CC-RIX mice were born between 12/31/2018 and 2/25/2019. Mice were shipped to Thomas Jefferson University Laboratory Animal Sciences Facility at approximately 5–6 weeks and given time to acclimate to the facilities before use in experiments. CC-RIX mouse lines, delivered in multiple shipments due to individual breeding schedules, were divided into 5 experiments. BALB/cByJ mice were added to each experiment as the reference strain. All mice were housed in micro-isolator boxes in specific pathogen free rooms under temperature, humidity and light cycle-controlled conditions. Mice received water ad libitum and fed rodent chow sterilized by autoclaving.

### Animal ethics

Protocols and procedures were conducted in compliance with ethical and regulatory standards for animal experimentation set by the National Institute of Health (NIH). The animal use protocol (00136) was approved by the Thomas Jefferson University Institutional Animal Care and Use Committee (IACUC). CC-RI and CC-RIX mice were produced by the Systems Genetics Core Facility at the University of North Carolina (UNC) (Animal Welfare Assurance #A3410-01). The animal use protocols for CC-RI mice (18–288) and for CC-RIX mice (17–285) were approved by the UNC IACUC. All animal use protocols adhere to the “Guide for the Care and Use of Laboratory Animals” published by the National Research Council, USA.

### Production of antigens

HIS-tagged recombinant *Ov-*103 was expressed as soluble protein in PichiaPink yeast and *Ov-*RAL-2 was expressed in *E. coli* BL21. Both vaccine antigens were expressed and purified as previously described^17^. Endotoxin was removed with a Q anion exchange column with less than 2.7 EU/mg in the final products.

### Immunization and challenge protocol

Immunization doses for each mouse consisted of 25 µg of each recombinant antigen, *Ov-*103 and *Ov*-RAL-2, mixed with Advax-2 (1 mg delta inulin plus 10 μg CpG55.2) (Vaxine Pty Ltd, Adelaide, South Australia) brought to a total volume of 100 µl using Tris-buffered saline (TBS) (Corning, Corning NY). Adjuvant control consisted of 1 mg Advax-2 brought to a total volume of 100 µl using TBS. On day 0, mice were immunized intramuscularly with Advax-2-formulated *Ov*-103/*Ov*-RAL-2 vaccine, or adjuvant control, by bilateral injection of 50 µl into each caudal thigh. All mice were boosted twice with either adjuvant control or adjuvant with the two antigens, at two-week intervals. Two weeks after the final immunization, all mice were challenged with 25 L3 in a diffusion chamber surgically implanted in a subcutaneous pocket on the rear flank of each mouse. The diffusion chambers were constructed with 14 mm Lucite rings covered with 5.0 µM pore-size Durapore membranes (EMDMillipore, Billerca, MA) and fused together using an adhesive made of a 1:1 mixture of 1,2-dichloroethane (Fisher Scientific, Pittsburg, PA) and acryloid resin (Rohm and Haas, Philadelphia, PA). After assembly, diffusion chambers were sterilized using 100% ethylene oxide followed by 12 hours of aeration. The L3 were suspended in a 1:1 mixture of NCTC-135 and Iscove’s modified Dulbecco’s medium (Sigma, St. Lois, MO) with 100 U penicillin, 100 µg streptomycin (Corning), 100 µg gentamicin (EMDMillipore) and 30 µg of chloramphenicol (APP Pharmaceuticals LLC, Schaumburge, IL) per ml. These antibiotics were selected to control bacterial contaminants while having no effect on the endosymbiont *Wolbachia* or on parasite development^55–60^. The chambers were recovered 7 days post-challenge and the contents collected for analysis.

### Recovery of larvae from diffusion chambers

The contents of each diffusion chamber were observed under a microscope and the number of surviving larvae was determined. Percent reduction of the challenge larvae was calculated by: [(average worm survival in control mice – average worm survival in immunized mice) ÷ average worm survival in control mice] × 100.

### Cell preparation and flow cytometry analysis

Cells were collected from diffusion chambers recovered from the control and immunized mice. Erythrocytes were lysed using BD Pharm Lyse (BD Biosciences, San Jose, CA) and recovered cells were filtered using a 70-μm cell strainer and then washed using FACS buffer [DPBS (Corning), 3% BSA (Gemini Bio-Products), 5 mM ethylenediaminetetraacetic acid (Sigma-Aldrich)]. Cells were then stained at 4 °C in the dark for 30 minutes with a 100 μl total volume cocktail of the following antibodies: anti-B220 BUV396 (1:200), anti-CD3e BV711 (1:200), anti-CD8a PerCP5.5 (1:200), CD11c PE-Cy7 (1:400), anti-Ly6G PE (1:400), anti-CD19 BV786 (1:800), anti-F4/80 BV421 (1:800), anti-Ly6C AF700 (1:800), anti-NK1.1 FITC (1:800), anti-CD4 V650 (1:1600), anti-CD11b PE-TxRed (1:1600) (BD Biosciences). The stained cells were washed and resuspended in 200 μl FACS buffer with 50 μl of CountBright absolute counting beads (Invitrogen, Waltham, MA) to determine exact cell numbers within the diffusion chambers. Analysis of cell profiles was conducted on a BD LSRFortessa (BD Biosciences) and the data was analyzed using FlowJo v10 (FlowJo LLC, Ashland, OR). Cell populations were determined using a gating strategy that eliminates dead cells, debris and doublets to identify T cells (CD3^+^), B cells (CD19^+^)^61^, NK cells (CD3^−^, CD19^−^, NK 1.1^+^)^62^, dendritic cells (CD3^−^, CD19^−^, NK1.1^−^, CD11c^+^, CD11b^+^), neutrophils (CD3^−^, CD19^−^, NK1.1^−^, Ly6G^+^, CD11b^+^,CD11c^−^), eosinophils (CD3^−^, CD19^−^, NK1.1^−^, CD11c^−^, Ly6G^−^, CD11b^+^, Ly6C^+^, SSC^Hi^), monocytes (CD3^−^, CD19^−^, NK1.1^−^, CD11c^−^, Ly6G^−^, CD11b^+^, SSC^lo^, Ly6C^lo^, F4/80^lo^) and macrophages (CD3^−^, CD19^−^, NK1.1^−^, CD11c^−^, Ly6G^−^, CD11b^+^, SSC^lo^, Ly6C^lo^, F4/80^hi^)^63^ (Supplementary Fig. 1).

### Cytokine and chemokine analysis of fluid recovered from diffusion chambers

Fluid recovered from the diffusion chambers from each mouse was analyzed using Milliplex map Kit magnetic bead panels per manufacturer’s protocols (EMDMillipore) and a MAG-PIX Luminex multiplexing instrument (Luminex, Austin, TX). Diffusion chamber fluid was analyzed for 11 cytokines (IFN-γ, TNF-α, IL-4, IL-5, IL-6, IL-9, IL-10, IL-13, IL-17A, GM-CSF and G-CSF) and 9 chemokines (eotaxin, RANTES, KC, MCP-1, IP-10, MIG/CXCL9, MIP-1α, MIP-1β and MIP-2). Analyte concentrations were calculated using Milliplex Analyst software (EMDMillipore).

### Cytokine analysis of antigen-stimulated spleen cell supernatants

Spleens were collected aseptically at the termination of the experiment, kept on ice in 0.1% BSA and homogenized into a single cell suspension. Following lysis of erythrocytes using sterile distilled cold water, the suspension was filtered through a 70 μm cell strainer and washed using Dulbecco’s Modified Eagle medium (Corning). Cells were cultured in flat-bottom 96-well plates, with each well containing 2×10^6^ cells. The cells were stimulated with 10 μg of either *Ov-*103 or *Ov-*RAL-2 for 72 hours at 37 °C. IL-4 uptake by splenocytes was blocked by addition of 0.5 μl of anti-IL-4R (BD Biosciences) to each well. Supernatants were collected following incubation and frozen at −20 °C. Cytokines in the supernatants were analyzed using Milliplex map Kit magnetic bead panels per manufacturer’s protocols (EMDMillipore) and a MAG-PIX Luminex multiplexing instrument (Luminex, Austin, TX). Stimulated spleen cell supernatants were analyzed for 14 different cytokines: IFN-γ, IL-2, IL-4, IL-5, IL-6, IL-10, IL-13, IL-21, IL-23, IL-27, IL-33, IL-17A, IL-17E and IL-17F. Analyte concentrations were calculated using Milliplex Analyst software (EMDMillipore).

### Antigen-specific antibody analysis of serum

Serum collected from each mouse at the termination of the experiment was tested by enzyme-linked immunosorbent assay (ELISA) for antigen-specific IgG1, and combined IgG2a, IgG2b and IgG2c (IgG2a/b/c) responses. Nunc MaxiSorp 96-well ELISA plates (Thermo Fisher) were pre-coated with 1 μg/ml coating protein (*Ov-*103 or *Ov-*RAL-2) in 0.1 M Carbonate buffer pH 9.6 and left overnight at 4 °C. All wash steps used 1x TBS + 0.05% Tween 20 (TBS-T). Wells were blocked using blocking buffer (2% BSA in 1x TBS-T) for 1 hour at approximately 21 °C and serum samples serially diluted in blocking buffer, using an appropriate starting dilution for each antigen and antibody subclass, and incubated for 90 minutes at 37 °C. Following incubation, secondary horseradish peroxidase-conjugated antibodies, goat anti-mouse IgG1 (1:15,000) or anti-IgG2a, IgG2b and IgG2c (1:15,000 each) (Southern Biotech, Birmingham, AL) were added for 45 minutes at approximately 21 °C. The reaction was developed by adding 100 μl of TMB solution (SeraCare, Gaithersburg, MD) for 10 minutes for the IgG1 assays or 15 minutes for the IgG2a/b/c assays. The reactions were stopped by adding 100 μl of TMB stop solution (SeraCare), and the optical densities (OD) were measured at 450 nm using an iMark plate reader (BioRad, Hercules, CA). Endpoint titers were calculated using SoftMax Pro software (Molecular Devices, San Jose, CA) with minimum positive titers determined by the lowest serum dilution from vaccinated mice with OD values three times higher than background.

### Statistics

Data for parasite survival was analyzed by multifactorial analysis of variance ANOVA with post-hoc Fisher’s least significant difference testing in Systat v.11 (Systat Inc., Evanstown, IL). The correlations between individual immune factors and the percentage recovery of larvae were analyzed with the Pearson correlation coefficient by strain/line. Each factor was log-transformed after the value of one added to the original value before calculating the correlation coefficients. P-values for the correlation coefficients were adjusted for multiple testing to control the false discovery rate (FDR)^64^. Cell counts, chemokine and cytokine content in the diffusion chambers, levels of cytokines in antigen-stimulated spleen cell supernatants, and the antigen-specific antibody responses were rank-transformed prior to analysis. Each immunological factor was analyzed separately using a mixed effects model that simultaneously evaluated the effect of vaccination within each line of CC-RIX mice. Fixed effects included terms for vaccination within each strain/line while the main effect of strain/line was treated as a random effect. Correlation among the strain/lines was modeled using a Toeplitz covariance structure for the random effect. P-values for the vaccine-induced effects within each strain/line were calculated for each immunological factor and subsequently adjusted for multiple testing to control the false discovery rate as described^64^. A heat map was created as a visual summary of the FDR-adjusted p-values of the vaccine-induced immunological factors induced within all strain/lines for all the factors. All analyses were completed using SAS 9.4 and SAS/STAT 15.1 (SAS Institute, Cary, NC).

### Reporting summary

Further information on research design is available in the Nature Research Reporting Summary linked to this article.

## Supplementary information

Supplementary Information

Reporting Summary

## Data Availability

The data that support the findings of this study are available in the article and the supplementary figures and tables as well as from the corresponding author upon request.
