# Supplementary material for: Onchocerca volvulus bivalent subunit vaccine induces protective immunity in genetically diverse collaborative cross recombinant inbred intercross mice
Source: NPJ Vaccines. 2021 Jan 26;6:17. doi: 10.1038/s41541-020-00276-2 (PMC7838260; doi:10.1038/s41541-020-00276-2)
Supplement: Supplementary file 1 — Supplementary Information [file 41541_2020_276_MOESM1_ESM.pdf]

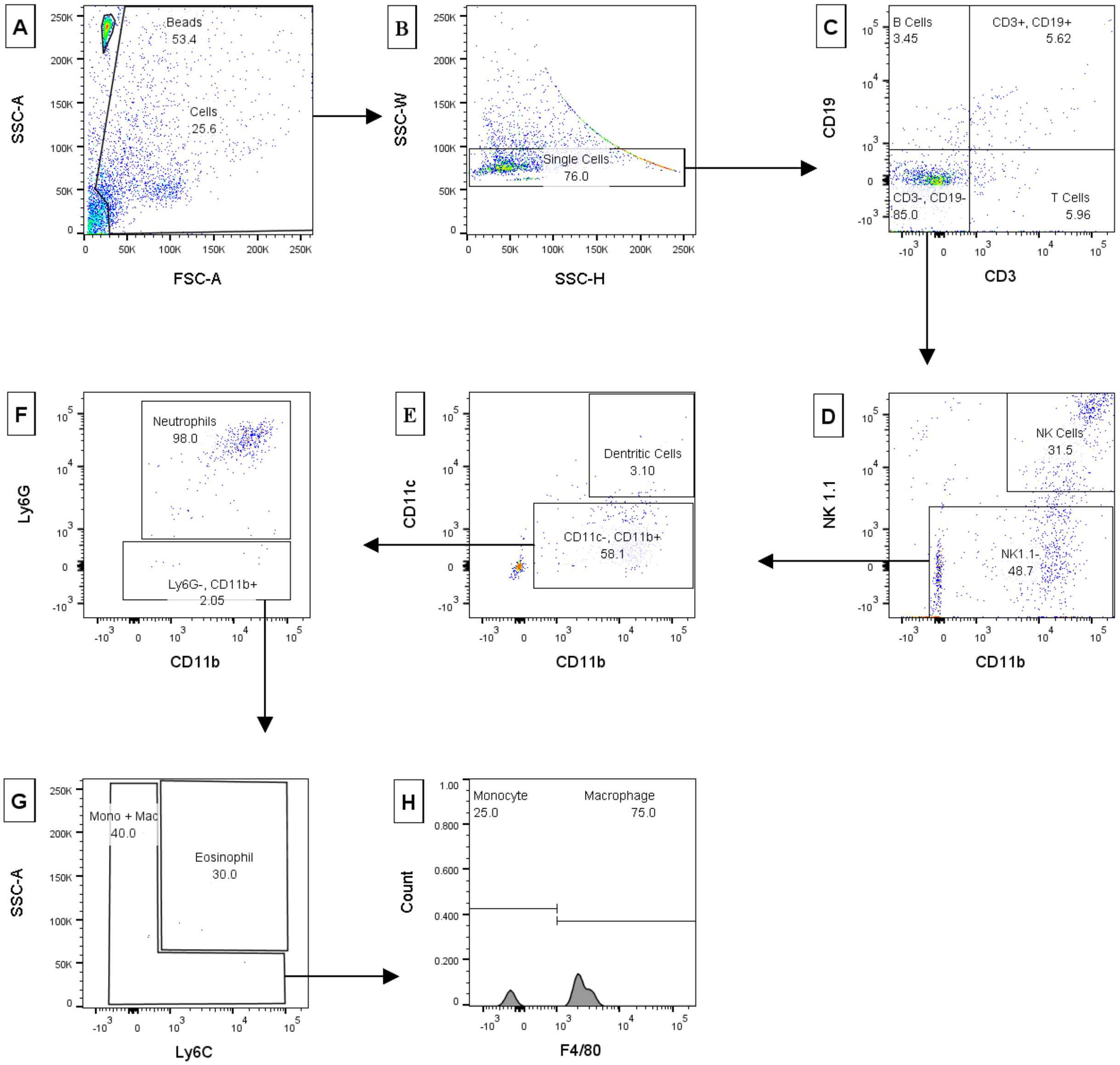

Supplementary Figure 1. Gating strategy for flow cytometry analysis. The protocol used to determine exact counts of cells within the diffusion chamber fluid. Dead cells and debris are gated out and a separate gate for CountBright beads was made (A). Doublets were gated out (B). $CD19^+$ B cells and $CD3^+$ T cells were gated and $CD3^-$, $CD19^-$ cells were selected (C). NK 1.1^+ natural killer cells were gated (D). $NK1.1^-$, $CD11c^+$, $CD11b^+$ dendritic cells were gated (E). $Ly6G^+$, $CD11b^+$, $CD11c^-$ neutrophils were gated (F). $Ly6G^-$, $CD11b^+$, $Ly6C^+$, SSC^{Hi} eosinophils were gated (G) and the remaining $F4/80^{Lo}$ monocytes and $F4/80^{Hi}$ macrophages were gated (H).

Strain	Sex	n	Treatment	Mean % Survival ± SD	p value
**BALB/cByJ**	Male	44	Control	74 ± 12	0.000
		45	Immune	42 ± 13	
**A** (CC004xCC071)	Male	4	Control	44 ± 6	0.383
		6	Immune	35 ± 18	
	Female	5	Control	43 ± 20	0.379
		5	Immune	47 ± 8	
**B** (CC005xCC001)	Male	4	Control	76 ± 12	0.000
		6	Immune	35 ± 9	
	Female	6	Control	62 ± 6	0.007
		5	Immune	38 ± 6	
**D** (CC019xCC055)	Male	4	Control	84 ± 9	0.000
		6	Immune	38 ± 7	
	Female	6	Control	59 ± 15	0.008
		4	Immune	35 ± 11	
**F** (CC039xCC003)	Male	6	Control	72 ± 3	0.007
		6	Immune	50 ± 16	
	Female	4	Control	77 ± 15	0.002
		5	Immune	47 ± 12	
**R** (CC040xCC002)	Male	6	Control	61 ± 20	0.001
		5	Immune	26 ± 8	
	Female	4	Control	47 ± 5	0.405
		7	Immune	39 ± 14	
**M** (CC042xCC007)	Male	4	Control	82 ± 15	0.000
		6	Immune	43 ± 7	
	Female	6	Control	57 ± 19	0.007
		4	Immune	31 ± 7	
**H** (CC051xCC049)	Male	4	Control	76 ± 15	0.000
		10	Immune	42 ± 10	
	Female	5	Control	78 ± 8	0.029
		2	Immune	56 ± 17	
**W** (CC026xCC006)	Male	6	Control	63 ± 12	0.033
		7	Immune	46 ± 10	
	Female	4	Control	77 ± 13	0.005
		3	Immune	49 ± 14	

Supplementary Table 1 - Survival of larval *Onchocerca volvulus* in vaccinated and challenged male and female CC-RIX and BALB/cByJ mice. Male and female mice were vaccinated intramuscularly with *Ov*-103 and *Ov*-RAL-2 formulated with Advax-2 while control mice received only Advax-2. Diffusion chambers containing challenge infections of 25 *O. volvulus* infective third-stage larvae were recovered one-week after implantation and larval survival was determined. Mean percent larval survival ± standard deviations are presented for each group. A $p \leq 0.05$ is considered to be statistically significant.
