# Supplementary material for: Onchocerca volvulus bivalent subunit vaccine induces protective immunity in genetically diverse collaborative cross recombinant inbred intercross mice
Source: NPJ Vaccines. 2021 Jan 26;6:17. doi: 10.1038/s41541-020-00276-2 (PMC7838260; doi:10.1038/s41541-020-00276-2)
Supplement: Supplementary file 2 — Reporting Summary [file 41541_2020_276_MOESM2_ESM.pdf]

nature research

Corresponding author(s): DBPR NPJVACCINES-00962R D. Abraham

Last updated by author(s): Dec 4, 2020

Reporting Summary

Nature Research wishes to improve the reproducibility of the work that we publish. This form provides structure for consistency and transparency in reporting. For further information on Nature Research policies, see our Editorial Policies and the Editorial Policy Checklist.

Statistics

For all statistical analyses, confirm that the following items are present in the figure legend, table legend, main text, or Methods section.

n/a	Confirmed	
☐	☒	The exact sample size (*n*) for each experimental group/condition, given as a discrete number and unit of measurement
☐	☒	A statement on whether measurements were taken from distinct samples or whether the same sample was measured repeatedly
☐	☒	The statistical test(s) used AND whether they are one- or two-sided<br>*Only common tests should be described solely by name; describe more complex techniques in the Methods section.*
☒	☐	A description of all covariates tested
☐	☒	A description of any assumptions or corrections, such as tests of normality and adjustment for multiple comparisons
☐	☒	A full description of the statistical parameters including central tendency (e.g. means) or other basic estimates (e.g. regression coefficient) AND variation (e.g. standard deviation) or associated estimates of uncertainty (e.g. confidence intervals)
☐	☒	For null hypothesis testing, the test statistic (e.g. *F*, *t*, *r*) with confidence intervals, effect sizes, degrees of freedom and *P* value noted<br>*Give P values as exact values whenever suitable.*
☒	☐	For Bayesian analysis, information on the choice of priors and Markov chain Monte Carlo settings
☒	☐	For hierarchical and complex designs, identification of the appropriate level for tests and full reporting of outcomes
☒	☐	Estimates of effect sizes (e.g. Cohen's *d*, Pearson's *r*), indicating how they were calculated

Our web collection on statistics for biologists contains articles on many of the points above.

Software and code

Policy information about availability of computer code

Data collection	na
Data analysis	na

For manuscripts utilizing custom algorithms or software that are central to the research but not yet described in published literature, software must be made available to editors and reviewers. We strongly encourage code deposition in a community repository (e.g. GitHub). See the Nature Research guidelines for submitting code & software for further information.

Data

Policy information about availability of data

All manuscripts must include a data availability statement. This statement should provide the following information, where applicable:

- Accession codes, unique identifiers, or web links for publicly available datasets
- A list of figures that have associated raw data
- A description of any restrictions on data availability

The data that support the findings of this study are available in the article and the supplementary figures and tables as well as from the corresponding author upon request.

Field-specific reporting

Please select the one below that is the best fit for your research. If you are not sure, read the appropriate sections before making your selection.

☒ Life sciences ☐ Behavioural & social sciences ☐ Ecological, evolutionary & environmental sciences

For a reference copy of the document with all sections, see nature.com/documents/nr-reporting-summary-flat.pdf

Life sciences study design

All studies must disclose on these points even when the disclosure is negative.

Sample size	Sample size was determined after a consultation with statisticians regarding previous data using the experimental procedures used in this study.
Data exclusions	na
Replication	All data is represented as two replicate experiments.
Randomization	Animals were assigned to experimental groups randomly.
Blinding	All experimental data was collected in a blinded manner. At the conclusion of the experiment codes were broken and data analyzed.

Reporting for specific materials, systems and methods

We require information from authors about some types of materials, experimental systems and methods used in many studies. Here, indicate whether each material, system or method listed is relevant to your study. If you are not sure if a list item applies to your research, read the appropriate section before selecting a response.

Materials & experimental systems

n/a	Involved in the study	
☐	☒	Antibodies
☒	☐	Eukaryotic cell lines
☒	☐	Palaeontology and archaeology
☐	☒	Animals and other organisms
☒	☐	Human research participants
☒	☐	Clinical data
☒	☐	Dual use research of concern

Methods

n/a	Involved in the study	
☒	☐	ChIP-seq
☐	☒	Flow cytometry
☒	☐	MRI-based neuroimaging

Antibodies

Antibodies used

Source Marker Clone Color Cat # LOT #
BD Biosciences CD45R/B220 RAS-6B2 BUV395 563793 8269770
BD Biosciences CD3e 145-2C11 BV711 563123 7311597
BD Biosciences CD8a 53-6.7 PerCP-Cy 5.5 551162 5345668
BD Biosciences CD11b M1/70 PE-CF594 562287 5051775
BD Biosciences CD11c HL3 PE-Cy 7 558079 4286714
BD Biosciences Ly-6G 1A8 PE 551461 5362993
BD Biosciences Ly-6C AL-21 AF700 561237 8270772
BD Biosciences NK-1.1 PK136 FITC 553164 3354935
BD Biosciences F4/80 T45-2342 BV421 565411 6091799
BD Biosciences CD19 1D3 BV786 563333 8250641
BD Biosciences CD4 RM4-5 PerCP5.5 563747 6154699
BD Biosciences Viability na BV510 564406 8316742

Southern Biotech goat anti-mouse IgG1 hrp 1073-05 A5312-N206C
Southern Biotech goat anti-mouse IgG2a hrp 1081-05 J0216-VH67B
Southern Biotech goat anti-mouse IgG2b hrp 1091-05 J1111-TI97B
Southern Biotech goat anti-mouse IgG2c hrp 1078-05 L3913-Y828C

Validation

Antibodies were validated by commercial laboratory from which they were purchased.

Animals and other organisms

Policy information about studies involving animals; ARRIVE guidelines recommended for reporting animal research

Laboratory animals	Male BALB/cByJ mice 6-8 weeks old were obtained from The Jackson Laboratory (Bar Harbor, Maine). Male and female CC-RIX mice: Line A – CC004/TauUnc x CC071/TauUnc; Line B – CC005/TauUnc x CC001/Unc; Line D- CC019/TauUnc x CC055/TauUnc; Line F – CC039/Unc x CC003/Unc; Line H – CC051/TauUnc x CC049/TauUnc; Line M – CC042/GeniUnc x CC007/Unc; Line R – CC040/TauUnc x CC002/Unc and Line W – CC026/GeniUnc x CC006/TauUnc. Ages 5-6 weeks at shipping, experiment start ~8 8 weeks old from System Genetics Core Facility at University of North Carolina (UNC), Chapel Hill
Wild animals	na
Field-collected samples	na
Ethics oversight	Protocols and procedures were conducted in compliance with ethical and regulatory standards for animal experimentation set by the National Institute of Health (NIH). The animal use protocol (00136) was approved by the Thomas Jefferson University Institutional Animal Care and Use Committee (IACUC). CC-RI and CC-RIX mice were produced by the Systems Genetics Core Facility at the University of North Carolina (UNC) (Animal Welfare Assurance #A3410-01). The animal use protocols for CC-RI mice (18-288) and for CC-RIX mice (17-285) were approved by the UNC IACUC. All animal use protocols adhere to the "Guide for the Care and Use of Laboratory Animals" published by the National Research Council, USA.

Note that full information on the approval of the study protocol must also be provided in the manuscript.

Flow Cytometry

Plots

Confirm that:

☒ The axis labels state the marker and fluorochrome used (e.g. CD4-FITC).

☒ The axis scales are clearly visible. Include numbers along axes only for bottom left plot of group (a 'group' is an analysis of identical markers).

☒ All plots are contour plots with outliers or pseudocolor plots.

☒ A numerical value for number of cells or percentage (with statistics) is provided.

Methodology

Sample preparation	Cells were collected from diffusion chambers recovered from the control and immunized mice. Erythrocytes were lysed using BD Pharm Lyse (BD Biosciences, San Jose, CA) and recovered cells were filtered using a 70-µm cell strainer and then washed using FACS buffer [DPBS (Corning), 3% BSA (Gemini Bio-Products), 5 mM ethylenediaminetetraacetic acid (Sigma-Aldrich)]. Cells were then stained at 4°C in the dark for 30 minutes with a 100 µl total volume cocktail of the following antibodies: anti-B220 BUV396 (1:200), anti-CD3e BV711 (1:200), anti-CD8a PerCP5.5 (1:200), CD11c PE-Cy7 (1:400), anti-Ly6G PE (1:400), anti-CD19 BV786 (1:800), anti-F4/80 BV421 (1:800), anti-Ly6C AF700 (1:800), anti-NK1.1 FITC (1:800), anti-CD4 V650 (1:1600), anti-CD11b PE-TxRed (1:1600) (BD Biosciences). The stained cells were washed and resuspended in 200 µl FACS buffer with 50 µl of CountBright absolute counting beads (Invitrogen, Waltham, MA) to determine exact cell numbers within the diffusion chambers
Instrument	BD LSRFortessa SOPR (BD Biosciences)
Software	FlowJo v10 (FlowJo LLC, Ashland, OR)
Cell population abundance	The samples were not sorted.
Gating strategy	Cell populations were determined using a gating strategy that eliminates dead cells, debris and doublets to identify T cells (CD3+), B cells (CD19+)61, NK cells (CD3-, CD19-, NK 1.1+)62, dendritic cells (CD3-, CD19-, NK1.1-, CD11c+, CD11b+), neutrophils (CD3-, CD19-, NK1.1-, Ly6G+, CD11b+,CD11c-), eosinophils (CD3-, CD19-, NK1.1-, CD11c-, Ly6G-, CD11b+, Ly6C+, SSCHi), monocytes (CD3-, CD19-, NK1.1-, CD11c-, Ly6G-, CD11b+, SSClo, Ly6Clo, F4/80lo) and macrophages (CD3-, CD19-, NK1.1-, CD11c-, Ly6G-, CD11b+, SSClo, Ly6Clo, F4/80hi)63 (Supplement, Figure 1).

☒ Tick this box to confirm that a figure exemplifying the gating strategy is provided in the Supplementary Information.
